# Social support helps protect against perinatal bonding failure and depression among mothers: a prospective cohort study

**DOI:** 10.1038/s41598-017-08768-3

**Published:** 2017-08-25

**Authors:** Masako Ohara, Takashi Okada, Branko Aleksic, Mako Morikawa, Chika Kubota, Yukako Nakamura, Tomoko Shiino, Aya Yamauchi, Yota Uno, Satomi Murase, Setsuko Goto, Atsuko Kanai, Tomoko Masuda, Masahiro Nakatochi, Masahiko Ando, Norio Ozaki

**Affiliations:** 1 Department of Psychiatry, Nagoya University Graduate School of Medicine, Nagoya, Japan; 2 Sugiyama Jogakuen University, Nagoya, Japan; 3 Graduate School of Education and Human Development, Nagoya University, Nagoya, Japan; 4 Graduate School of Law, Nagoya University, Nagoya, Japan; 5 Center for Advanced Medicine and Clinical Research, Nagoya University Hospital, Nagoya, Japan

## Abstract

Causal relationships between perinatal bonding failure, depression, and social support among mothers remain unclear. A total of 494 women (mean age 32.4 ± 4.5 years) completed the Mother-Infant Bonding Questionnaire (MIBQ), the Edinburgh Postnatal Depression Scale (EPDS), and the Japanese version of the Social Support Questionnaire in early pregnancy before week 25 (T1) and 1 month after delivery (T2). Our model of recursive structured equation modeling (SEM) showed acceptable fit (CMIN/*df* = 2.2, CFI = 0.97, and RMSEA = 0.05). It was revealed that: (1) a lower number of supportive persons at T1 significantly predicted both MIBQ and EPDS scores at T1 and T2; (2) at T1, poorer satisfaction with the social support received significantly predicted EPDS scores; (3) both MIBQ and EPDS scores at T1 significantly predicted their respective scores at T2. Out cohort study indicates that the number of individuals who are available to provide social support and the degree of satisfaction with the level of social support received during pregnancy have a great influence on bonding failure and depression in the postpartum period. These findings suggest that psychosocial interventions that focus on these two aspects of social support during pregnancy are effective in preventing bonding failure and depression in the postpartum period.

## Introduction

The difficulties that some mothers have in establishing an emotional bond with their newborns, often referred to as bonding failure, are an important focus in perinatal psychiatry^1, 2^. In his review of perinatal psychiatry research and practice in recent times, Brockington^3^ stated that mother-infant relationship disorders are specific conditions, which are not caused by a single entity, but rather, are a group of overlapping clinical states with various morbid elements in the relationship between the mother and infant^3^. The manifestations of bonding failure include lack of maternal affective involvement, increased irritability, aggressive impulses, or outright rejection of the infant^3^. These factors may result in abusive parenting^4, 5^, insecure interactions between the mother and infant^6^, and disturbance of early childhood development^7^. Therefore, it is important to identify predictive factors for bonding failure and to intervene at an early stage from the standpoint of the mental health care of mothers.

Previous studies have investigated psychopathological or psychosocial factors that may be associated with bonding failure. Bonding failure often coexists with maternal depression^8^. Nevertheless, most studies indicating a link between bonding failure and depression were cross-sectional^9–11^. Therefore, causality has not been determined. Two longitudinal studies showed that postpartum depression had a significant impact on maternal bonding^12, 13^. However, the studies used regression analysis. Therefore, it remains unclear if maternal depression leads to bonding failure or vice versa, or if the two factors are confounded by a third variable or different mechanism. Thus, it is essential to conduct a prospective study that incorporates causal path models. The first research question of the present research is the possible causative links between perinatal bonding failure and depression as well as search for potential confounding factors.

Social support has been studied as a key to understanding psychological adjustment and maladjustment. In a situation where individuals are exposed to stressful events, social support protects them from potentially adverse effects of stressful events^14^. Social support is usually measured in terms of the number of resource persons available and the perceived quality of the support received. It was reported that psychological maladjustment would occur in stressful situations when the perceived quality (satisfaction) of support was poor^15^. The association between depression and social support has been investigated in many previous studies. Poor perceived support was reported to be related to depression among women in the perinatal period. However, one longitudinal study showed that having a lower number of supportive persons during pregnancy predicted postpartum depression^16^.

Bonding failure may be linked to social support. However, very few studies have investigated this issue. Kitamura *et al*.^17^ used regression analysis to examine the influence of social support on bonding failure in the postpartum period^17^. They showed that poor satisfaction with support at baseline and disappointment due to the absence of expected support after childbirth were direct causes of abusive parenting. However, that study examined social support and bonding failure simultaneously in the postpartum period. Therefore, it remains unclear whether a lack of social support during pregnancy predicts bonding failure in the postpartum period. To address this issue, it is essential to assess bonding failure and social support prospectively during pregnancy and the postpartum period.

Recently, Ohashi *et al*.^18^ used regression analysis to examine whether bonding difficulty after childbirth was predicted by poor satisfaction with hospital care during pregnancy and the postpartum period^18^. They showed that mothers’ anger and rejection towards their infants could be explained by perceived lack of satisfaction with medical and nursing care; however, social support was evaluated only by mothers’ satisfaction with hospital care. It is essential to evaluate social support from family, friends, and significant others as well as hospital care. The second goal of this study was to determine the potential link among social support, perinatal bonding failure, and depression. We were particularly interested in whether quantity or quality of social support was important in understanding perinatal mental health and if these factors have different relationships with perinatal bonding failure and depression.

A prospective cohort study is required to address the causal relationships between the three variables of perinatal bonding failure, depression, and social support. Path models allow researchers to posit causality from one variable to another. Structured equation modeling (SEM) with goodness-of-fit indices enables researchers to compare path models in terms of how well the models fit the data. Such a comparison of models may lead to a better cue to presume the time sequence of variables.

Therefore, the aim of our study was to examine prospectively whether bonding failure and depression in the postpartum period are predicted by the number of individuals who are available to provide social support and/or the degree of satisfaction with the level of social support received during pregnancy.

## Materials and Methods

### Ethics Statement

The study was explained to all participants both verbally and in writing, and written informed consent was obtained from each participant. This study protocol was approved by the Ethics Committee of the Nagoya University Graduate School of Medicine, the Ethics Committee of Kaseki Hospital, and the Ethics Committee of Nagoya Teishin Hospital. The study was conducted in accordance with the established ethical standards of all institutions. The authors assert that all procedures contributing to this work complied with the ethical standards of the relevant national and institutional committees on human experimentation and with the Helsinki Declaration of 1975, as revised in 2008.

### Participants

Participants in this study were recruited from perinatal classes for pregnant women (starting before week 25 of pregnancy) at two obstetric hospitals and one university hospital in central Nagoya, Japan (with a population of approximately 2 million) between August 2004 and November 2015. Mothers with current or past histories of mental illness were excluded from the study, as well as mothers with children born before week 32 of gestation. In addition, participants were required to be at least 20 years old and capable of understanding the Japanese language.

### Procedures

**Figure 1:**
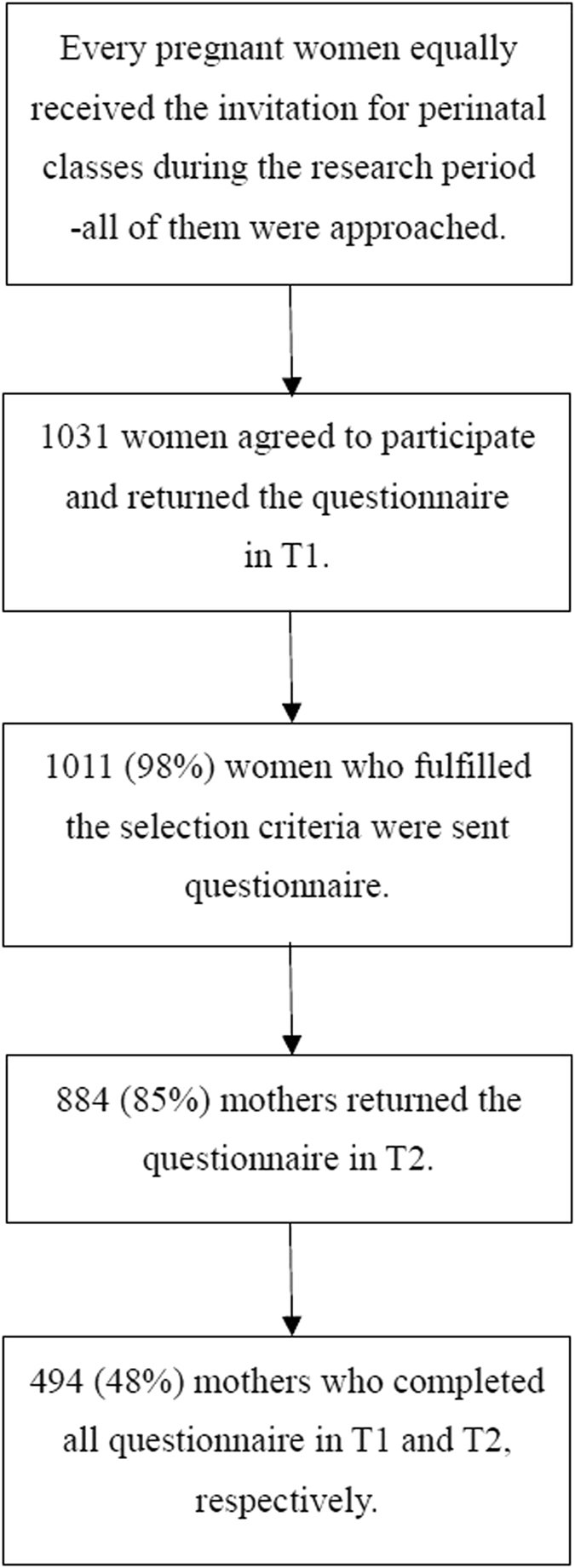
Flow chart of the mothers’ participation rate. Abbreviations: T1, early pregnancy before week 25; T2, 1 month after delivery.

Pregnant women attending perinatal classes who agreed to participate in the study were asked to complete self-reporting questionnaires in early pregnancy before week 25 (T1) and to return them by mail. Questionnaires consisted of the Mother-Infant Bonding Questionnaire (MIBQ), the Edinburgh Postnatal Depression Scale (EPDS), the J-SSQ, and social demographic questions such as age, parity (primipara/multipara), number of children, and partner’s age. After the completed consent forms and questionnaires were received, the MIBQ and the EPDS were sent to participants again at 1 month after delivery (T2) and were again returned by mail. In the current study, we used the scores of the MIBQ, the EPDS, and the J-SSQ at T1, and those of the MIBQ and the EPDS at T2. A total of 1031 women agreed to participate in the perinatal classes starting before week 25 of pregnancy; 1011 (98%) women fulfilled the selection criteria; 494 mothers (48%; mean age, 32.4 ± 4.5 years) completed all questionnaires necessary for the analysis (Fig. 1).

### Measures

#### Mother-Infant Bonding Questionnaire (MIBQ)

The MIBQ is a self-reporting scale designed to assess maternal bonding of mothers with their babies during the postpartum period. It is composed of nine items with responses rated on a four-point Likert scale (from 0, “very much” to 3, “not at all”), with the scale of some items being reversed^19, 20^. Total scores range from 0 to 27. A high score indicates worse mother-to-infant bonding.

In our previous study, we reported the reliability and validity of the MIBQ in the pregnancy and postpartum periods. In addition, we examined the factor structure of the MIBQ and demonstrated a two-factor model: “Lack of Affection” (LA) and “Anger and Rejection” (AR)^20^.

#### Edinburgh Postnatal Depression Scale (EPDS)

The EPDS is a self-reporting questionnaire designed to assess postpartum depression; it is composed of 10 items scored on a four-point Likert Scale (0 to 3) yielding a total score ranging from 0 to 30^21^. Numerous studies have used this instrument during the pregnancy and postpartum periods. The Japanese version of the EPDS showed good internal consistency (Cronbach’s alpha = 0.78) and test-retest reliability (Spearman’s correlation = 0.92)^22^. A score ≥ 9 was designed to screen for minor and major depressive episodes, with a sensitivity of 75% and 82% and a specificity of 93% and 95%, respectively^22, 23^. Our colleagues examined the factor structure of the EPDS and demonstrated a three-factor model: “Anxiety”, “Depression”, and “Anhedonia”^24^.

#### Japanese version of the Social Support Questionnaire (J-SSQ)

The J-SSQ, which was standardized by Furukawa *et al*.^25^, is the Japanese version of the SSQ6^25^. The SSQ6^26^ is a brief measure of social support based on the long form (27 items) of the SSQ^27^. The SSQ6 is composed of 12 items. Our colleagues examined the reliability and validity of the J-SSQ in the pregnancy and postpartum periods, and demonstrated a two-factor model: “Number of Persons” (NP) and “Satisfaction Rating” (SR)^16^. NP reflects the sum of the perceived number of available others who provide social support as measured by the total score on the Number subscale of the J-SSQ. SR reflects the sum of the individual’s degree of satisfaction with the perceived support available in each situation, as measured by the total score on the Satisfaction subscale of the J-SSQ.

#### Statistical analysis

We first calculated descriptive statistics for the MIBQ, EPDS and J-SSQ. We log-transformed all MIBQ and EPDS scores that were positively skewed for the subsequent analysis. As for the J-SSQ, the scores on the Number subscale were log-transformed for the subsequent analysis due to positively skewed distributions. We calculated the means and standard deviations of all variables used in this study and then correlated them. From there, we created a path model with MIBQ subscales (LA and AR), EPDS subscales (Anxiety, Depression, and Anhedonia), and J-SSQ subscales (NP and SR) as predictors to clarify the relationships between the variables. Here, we created two latent variables (bonding failure and depressive mood) at each observation point. Bonding failure consists of the two MIBQ subscales: LA and AR whereas depression consists of the EPDS subscales: Anxiety, Depression, and Anhedonia. In our path model, we posited that (1) NP in T1 would predict bonding failure in T1 and T2; (2) SR in T1 would predict bonding failure in T1 and T2; (3) similarly, NP in T1 would predict depression in T1 and T2; (4) SR in T1 would predict depression in T1 and T2; (5) bonding failure would predict depression at the next observation point whereas depression would predict bonding failure at the next observation point; (6) both bonding failure and depression at each observation point would predict its own counterpart at the next observation point, and (7) in both T1 and T2, bonding failure and depression are correlated with each other, and we therefore set covariances between their error variables. Therefore, our model was recursive.

The fit of the model with the data was examined in terms of chi-squared/degree of freedom (CMIN/*df*), comparative fit index (CFI), and root mean square error of approximation (RMSEA). According to conventional criteria, a good fit is indicated by CMIN/*df* < 2, CFI > 0.97, and RMSEA < 0.05, while CMIN/*df* < 3, CFI > 0.95, and RMSEA <0.08 demonstrate an acceptable fit^28^. To compare all the models and to determine the best model to fit the data, we used the Akaike Information Criterion (AIC). A model with an AIC value at least two points lower than that of a competing model is regarded as a better model^29^.

All statistical analyses were conducted using the SPSS version 22.0 and Amos 21.0 (IBM Japan, Tokyo, Japan).

## Results

### Demographics

The mean (SD) age of the participants was 32.4 (4.5) years. There were 391 first-time mothers (79%) and 94 multiparous (9 did not specify). Demographic characteristics of the study participants, including age, number of children, and partner’s age, did not statistically significantly differ from women who were excluded from the study (Table 1). In addition, these statistics did not differ from those of the whole mother’s group (see Supplementary Table S1).

**Table 1:** Demographic characteristics of included and excluded mothers

	Included mothers (n = 477–494) Mean (SD)		Excluded mothers (n = 496–517) Mean (SD)		*t*	*p*
Age (years)	32.4	(4.5)	31.9	(4.5)	1.93	0.43
Number of children	0.24	(0.53)	0.26	(0.53)	0.72	0.30
Partner’s age (years)	34.7	(5.7)	34.4	(5.8)	0.74	0.45

### Correlations of the variables used in this study

The means and standard deviations of all the variables used in this study and their correlations are shown in Table 2. NP was negatively correlated with both the MIBQ and the EPDS subscales over both periods. SR was negatively correlated with the EPDS subscales in T1, Depression in T2, and AR in T2. In all periods, the correlations between NP and other subscales were larger than those of SR and other subscales. The scores of LA were correlated over both periods. Those of AR were also correlated over both periods. Similarly, all EPDS subscales were correlated over both periods. The MIBQ subscales in T1 were correlated with the EPDS subscales in T2. Depression in T1 was correlated AR in T2. In T1, the MIBQ subscales were correlated with the EPDS subscales, except for the correlation between LA and Anxiety. In T2, the MIBQ subscales were correlated with the EPDS subscales. Changes of the variables are shown in Table 3. The scores of LA decreased from T1 to T2 (t = 15.72, *p* < 0.01). The scores of AR and ANH increased from T1 to T2 (t = 7.12, *p* < 0.01; t = 5.00, *p* < 0.01).

**Table 2:** Means and SDs of and correlations^a^ between the variables used in this study.

	1	2	3	4	5	6	7	8	9	10	11	12
1.NP; T1												
2. SR; T1	0.17^**^											
3. LA; T1	−0.10^*^	−0.08										
4. AR; T1	−0.09^*^	−0.09	0.20^**^									
5. ANX; T1	−0.15^**^	−0.10^*^	0.08	0.12^**^								
6. DEP; T1	−0.25^**^	−0.13^**^	0.14^**^	0.18^**^	0.60^**^							
7. ANH; T1	−0.22^**^	−0.18^**^	0.18^**^	0.21^**^	0.33^**^	0.47^**^						
8. LA; T2	−0.10^*^	−0.08	0.45^**^	0.08	0.02	0.07	0.07					
9.AR; T2	−0.18^**^	−0.11^*^	0.23^**^	0.31^**^	0.07	0.09^*^	0.07	0.23^**^				
10.ANX; T2	−0.23^**^	−0.02	0.11^*^	0.13^**^	0.46^**^	0.35^**^	0.21^**^	0.14^**^	0.26^**^			
11. DEP; T2	−0.22^**^	−0.11^*^	0.16^**^	0.17^**^	0.38^**^	0.41^**^	0.29^**^	0.26^**^	0.27^**^	0.65^**^		
12. ANH; T2	−0.12^*^	−0.09	0.14^**^	0.09^*^	0.19^**^	0.19^**^	0.26^**^	0.32^**^	0.25^**^	0.40^**^	0.54^**^	
Mean	8.83	29.82	1.82	0.08	1.63	0.67	0.15	0.80	0.24	1.56	0.75	0.28
SD	2.33	6.86	1.58	0.31	1.22	0.92	0.42	1.01	0.51	1.25	0.93	0.56
Skewness	0.41	−2.05	0.63	4.47	0.17	1.29	3.26	1.52	2.28	0.20	1.25	1.96
Alpha	0.89	0.97	0.76	0.58	0.75	0.77	0.78	0.63	0.62	0.78	0.76	0.86

^a^Pearson’s correlations.

^*^p < 0.05, ^**^p < 0.01.

Abbreviations: Alpha, Chronbach’s alpha; ANH, Anhedonia; ANX, Anxiety; AR, Anger and Rejection; DEP, Depression; LA, Lack of Affection;

NP, Number of Persons; SD, Standard deviation; SR, Satisfaction Rating; T1, early pregnancy before week 25; T2, 1 month after delivery.

**Table 3:** Changes of the variables (mean [SD]) assessed using the paired t-test

Variables	T1			T2 Difference between T1 and T2 (t)			
	Mean (SD)	Skewness	Skewness after log transformation	Mean (SD)	Skewness	Skewness after log transformation	t
Lack of Affection	1.82(1.58)	0.84	0.63	0.80(1.01)	1.60	1.52	15.72**
Anger and Rejection	0.08(0.31)	4.72	4.47	0.24(0.51)	2.74	2.28	7.12**
Anxiety	1.63(1.22)	0.52	0.17	1.56(1.25)	1.81	1.25	1.30
Depression	0.67(0.92)	1.70	1.29	0.75(0.93)	0.51	0.20	1.79
Anhedonia	0.15(0.42)	4.18	3.26	0.28(0.56)	2.79	1.95	5.00**

^**^ *p* < 0.01.

Abbreviations: T1, early pregnancy before week 25; T2, 1 month after delivery.

### Path model

**Figure 2:**
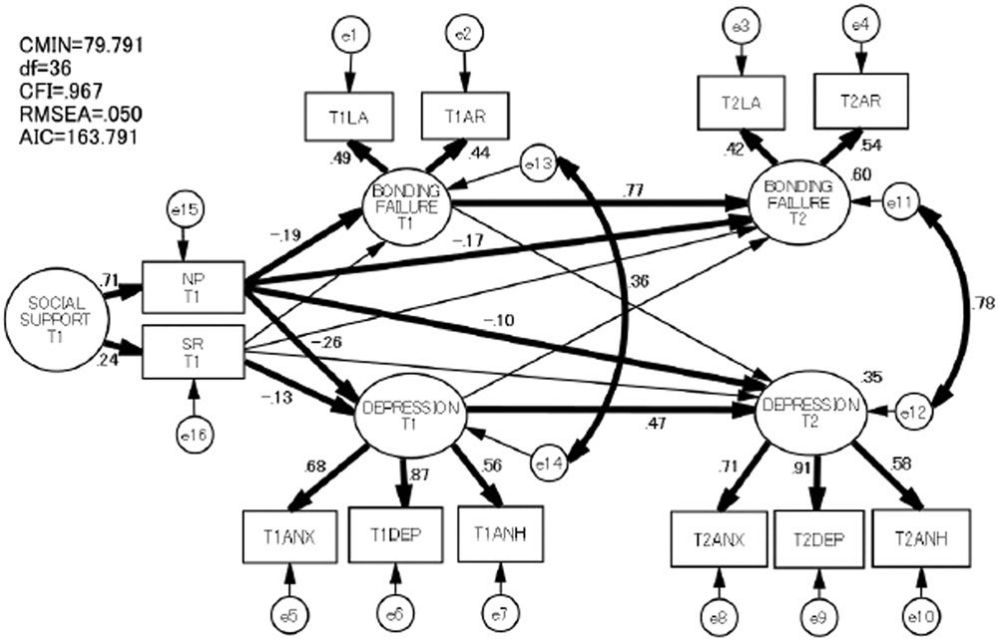
Path model of the association between bonding failure, depression, and social support during T1 and T2. Significant paths and covariances are shown in bold. Covariances between the indicators over the two periods are not shown for clarity. Abbreviations: AIC, Akaike Information Criterion; ANH, Anhedonia; ANX, Anxiety; AR, Anger and Rejection; CFI, comparative fit index; CMIN, chi-squared; DEP, Depression; df, degree of freedom; e, error variables; LA, Lack of Affection; NP, Number of Persons; RMSEA, root mean square error of approximation; SR, Satisfaction Rating; T1, early pregnancy before week 25; T2, 1 month after delivery.

Figure 2 shows our path model of recursive SEM, which showed reasonable fit with the data (CMIN/*df* = 2.2, CFI = 0.97, RMSEA = 0.05). The CMIN/*df* values indicated an acceptable model of fit. The fitness of the model to the data, the CFI, and the RMSEA, were acceptable for the data. This model found that: (1) NP in T1 predicted bonding failure in T1 and T2 (*p* < 0.01, r = −0.19; *p* < 0.01, r = −0.17); (2) NP in T1 predicted depression in T1 and T2 (*p* < 0.01, r = −0.26; *p* < 0.01, r = −0.10); (3) SR in T1 predicted depression in T1 (*p* < 0.05, r = −0.13); (4) bonding failure as well as depression predicted their counterpart at the next observation point (*p* < 0.01, r = 0.77; *p* < 0.01, r = 0.47); and (5) error variables between bonding failure and depression at each observation point were correlated (*p* < 0.01, r = 0.36; *p* < 0.01, r = 0.78). In T2, the determinant coefficients of bonding failure and depression were 0.60 and 0.35, respectively.

## Discussion

To the best of our knowledge, the present study is the first to investigate prospectively whether bonding failure and depression are predicted by the number of individuals who are available to provide social support and/or the degree of satisfaction with the level of social support received during pregnancy in a large cohort of pregnant women. We created a path model with the MIBQ subscales, EPDS subscales, and the J-SSQ subscales as predictors to clarify the causal relationships between perinatal bonding failure, depression, and social support, and the model was tested with SEM.

A unique finding of this study was the link between the number of supportive persons during pregnancy, bonding failure and depression in the postpartum period. Our path model showed a lower number of supportive persons during pregnancy directly predicted bonding failure in the postpartum period. In addition, our path model showed a lower number of supportive persons during pregnancy was a cause of bonding failure in the postpartum period due to increased bonding failure during pregnancy. Similarly, our path model showed a lower number of supportive persons directly predicted depression in the postpartum period. This is in line with a previous report that examined the association between social support during pregnancy and postpartum depressive state using multivariate liner regression analysis^16^. In addition, our path model showed a lower number of supportive persons during pregnancy was a cause of depression in the postpartum period due to increased depression during pregnancy. Therefore, this suggests that in clinical settings, we should pay more attention to the number of available supportive persons during pregnancy because of the direct/indirect link with depression and bonding failure in the postpartum period.

Another unique finding of this study was the link between satisfaction with the social support received during pregnancy and depression in the postpartum period. Our path model showed that satisfaction with the social support received during pregnancy did not directly predict depression in the postpartum period at a statistically significant level. However, poorer satisfaction with the social support received during pregnancy was a cause of depression in the postpartum period due to increased depression during pregnancy. Therefore, this suggests that in clinical settings, psychological interventions should focus on interpersonal support to prevent depression during pregnancy because of its indirect link with depression in the postpartum period.

Another important feature of this study is that the determinant coefficients of bonding failure and depression in the postpartum period were 0.60 and 0.35, respectively. This means that 60% of the variance of bonding failure and 35% of that of depression were explainable by this path model.

Error variables of bonding failure and depression shared variances at each time point. We speculated that these two issues often coexisted. However, our path model revealed that bonding failure and depression were causally independent. These results might suggest the presence of confounders that explain the coexistence of bonding failure and depression. For example, the association between personality and bonding failure has been investigated in previous studies. It has been reported that bonding failure was predicted by low self-directedness and co-operativeness^30^. Additionally, personality is one of the major factors associated with maternal depression. Minatani *et al*. reported that severity of depression during pregnancy was predicted by low self-directedness, high harm-avoidance, persistence, and self-transendence^31^. Such unidentified confounding factors were not evaluated in this study, therefore, further longitudinal studies are necessary to clarify this possibility.

One of the strengths of our study was its prospective method, which enabled us to unravel causal links between variables. When studies are cross-sectional, the results are correlational, so determining causality is almost impossible.

Our study results clarified the fact that two aspects of social support, number of persons and satisfaction with the social support received during pregnancy, have a great influence on bonding failure and depression in the postpartum period. These results indicate that psychosocial interventions that focus on the social support network during pregnancy are effective in preventive bonding failure and depression in the postpartum period. This finding points to the benefits of early interventions to increase the number of support providers for pregnant women. In addition, it is also important that professionals in clinical settings pay more attention to building supportive and sympathetic relationships with women and appropriately address individual satisfaction with social support during pregnancy.

## Limitations

Some limitations of this study should be noted. First, there are numerous variables known to be associated with bonding failure and depression, as well as potentially confounding variables related to pregnancy, delivery, and infants. Other potential factors include maternal age, family condition, mode of feeding^32^, and birth status (preterm or full-term)^33^. For example, mothers of very preterm infants report lower postnatal maternal bonding compared to full-term counterparts^33^. However, we did not examine those potentially important variables and factors in this study. Future investigations should incorporate this additional information. Second, the participants of this study were recruited from perinatal classes for pregnant women, and they participated in this study voluntarily. Therefore, this sample may not be representative of the total population. Additionally, depression and bonding failure were evaluated only with self-reports by the participants. Structured diagnostic interviews are preferable in order to reach a clinical diagnosis of depression. Another major limitation of this study was the attrition rate of the participants. Only 48% of eligible women completed the two-wave surveys. Caution should be exercised before conclusion partly because of log-transformation of data of the variables used in the SEM. There is no unanimous consensus on the need to transform the scale values when they are not normally distributed. In fact, some researchers believe that such transformations make it difficult to interpret the results. Hence, we also performed our data analyses without log-transformation, and found similar results.

## Conclusions

Our results indicate that the number of individuals who are available to provide social support and the degree of satisfaction with the level social support received during pregnancy have a great influence on bonding failure and depression in the postpartum period. These results suggest that psychosocial interventions that focus on these two aspects of social support during pregnancy are effective in preventing bonding failure and depression in the postpartum period.

## Electronic supplementary material

Supplementary Information
