# Supplementary material for: Social support helps protect against perinatal bonding failure and depression among mothers: a prospective cohort study
Source: Sci Rep. 2017 Aug 25;7:9546. doi: 10.1038/s41598-017-08768-3 (PMC5572740; doi:10.1038/s41598-017-08768-3)
Supplement: Supplementary file 1 — Supplementary Information [file 41598_2017_8768_MOESM1_ESM.pdf]

Social support helps protect against perinatal bonding failure and depression among mothers: a prospective cohort study – Supplementary Information

Masako Ohara[1)], Takashi Okada[1)*], Branko Aleksic[1)], Mako Morikawa[1)], Chika Kubota[1)], Yukako Nakamura[1)], Tomoko Shiino[1)], Aya Yamauchi[1)], Yota Uno[1)], Satomi Murase[1)], Setsuko Goto[2)], Atsuko Kanai[3)], Tomoko Masuda[4)], Masahiro Nakatochi[5)], Masahiko Ando[5)], and Norio Ozaki[1)]

1) Department of Psychiatry, Nagoya University Graduate School of Medicine, Nagoya, Japan
2) Sugiyama Jogakuen University, Nagoya, Japan
3) Graduate School of Education and Human Development, Nagoya University, Nagoya, Japan
4) Graduate School of Law, Nagoya University, Nagoya, Japan
5) Center for Advanced Medicine and Clinical Research, Nagoya University Hospital, Nagoya, Japan

*Corresponding author
Takashi Okada, M.D., Ph.D.
Department of Psychiatry, Nagoya University Graduate School of Medicine
65 Tsurumai-cho, Showa-ku, Nagoya, Aichi 466-8550, Japan
E-mail: okada@med.nagoya-u.ac.jp
Tel: +81-52-744-2282, Fax: +81-52-744-2293

Table of contents

Supplementary Table S1. Demographic characteristics of included and all mothers

Supplementary Table S1. Demographic characteristics of included and all mothers

	Included mothers (n= 477-494) Mean (SD)		all mothers (n= 973-1011) Mean (SD)		t	*p*
Age (years)	32.4	(4.5)	32.1	(4.5)	1.13	0.65
Number of children	0.24	(0.53)	0.25	(0.53)	0.42	0.54
Partner’s age (years)	34.7	(5.7)	34.5	(5.8)	0.43	0.68
